# A sandwich amperometric immunosensor for the detection of fowl adenovirus group I based on bimetallic Pt/Ag nanoparticle-functionalized multiwalled carbon nanotubes

**DOI:** 10.1038/s41598-023-50821-x

**Published:** 2024-01-02

**Authors:** Jiaoling Huang, Zhixun Xie, Sisi Luo, Meng Li, Liji Xie, Qing Fan, Tingting Zeng, Yanfang Zhang, Minxiu Zhang, Zhiqin Xie, Sheng Wang, Dan Li, You Wei, Xiaofeng Li, Lijun Wan, Hongyu Ren

**Affiliations:** Guangxi Key Laboratory of Veterinary Biotechnology, Key Laboratory of China (Guangxi)-ASEAN Cross-Border Animal Disease Prevention and Control, Ministry of Agriculture and Rural Affairs of China, Guangxi Veterinary Research Institute, Nanning, Guangxi China

**Keywords:** Biochemistry, Chemical biology

## Abstract

An enzyme-free sandwich amperometric immunosensor based on bimetallic Pt/Ag nanoparticle (Pt/AgNPs)-functionalized chitosan (Chi)-modified multiwalled carbon nanotubes (MWCNTs) as dual signal amplifiers and Chi-modified MWCNTs (MWCNTs-Chi) as substrate materials was developed for ultrasensitive detection of fowl adenovirus group I (FAdV-I). MWCNTs have a large specific surface area, and many accessible active sites were formed after modification with Chi. Hence, MWCNTs-Chi, as a substrate material for modifying glassy carbon electrodes (GCEs), could immobilize more antibodies (fowl adenovirus group I-monoclonal antibody, FAdV-I/MAb). Multiple Pt/AgNPs were attached to the surface of MWCNTs-Chi to generate MWCNTs-Chi-Pt/AgNPs with high catalytic ability for the reaction of H_2_O_2_ and modified active sites for fowl adenovirus group I-polyclonal antibody (FAdV-I/PAb) binding. Amperometric i–t measurements were employed to characterize the recognizability of FAdV-I. Under optimal conditions, and the developed immunosensor exhibited a wide linear range (10^0.93^ EID_50_ mL^−1^ to 10^3.43^ EID_50_ mL^−1^), a low detection limit (10^0.67^ EID_50_ mL^−1^) and good selectivity, reproducibility and stability. This immunosensor can be used in clinical sample detection.

## Introduction

Avian adenoviruses were separated into 3 groups (I–III). Group I, the so-called fowl adenovirus group I (FAdV-I), comprises 12 serotypes (FAdV-1, FAdV-2, FAdV-3, FAdV-4, FAdV-5, FAdV-6, FAdV-7, FAdV-8a, FAdV-8b, FAdV-9, FAdV-10, and FAdV-11) and mainly infects turkeys, chickens, ducks, geese and pigeons^1,2^. FAdV-I can be transmitted horizontally and vertically through feces and progeny, respectively^3^. It has been demonstrated that infections caused by FAdV-I are associated with hydropericardium, hepatitis and runting-stunting, resulting in apathy, weight loss, and mortality^4^. FAdV-I has been reported in many countries, and FAdV-I infections cause enormous economic burdens in poultry farming ^4,5^. Therefore, rapid, specific and sensitive detection methods are needed to screen for and control FAdV-I infection. Methods such as virus isolation and identification, polymerase chain reaction (PCR)-based assays and loop-mediated isothermal amplification (LAMP) have been proposed^6–9^. However, these diagnostic methods require either highly qualified personnel or sophisticated instrumentation.

Amperometric immunosensors are particularly useful for detecting various diseases because of their rapid detection, small analyte volume, high specificity and low detection limits for analyzing complex clinical samples with relatively simple instruments and procedures^10,11^. Amperometric immunosensors mainly include label-free and sandwich amperometric immunosensors. Label-free amperometric immunosensors, that is, direct immunosensors, work by measuring the signal changes arising directly from immune reactions without requiring labeling; these immunosensors are used for fast, real-time detection^12^. However, when other proteins or antigens are present in a clinical sample, nonspecific binding of the other proteins or antigens on the surface of the substrate can occur, and a small signal is generated, leading to an increase in the background signal, which results in a decrease in sensitivity^10^. In sandwich immunosensors, the antigen is sandwiched between the primary and detection antibodies. The primary antibody, which is immobilized on a solid substrate surface and used to capture the antigen from the sample, is known as the capture antibody. Detection antibodies, known as labeled antibodies, are used to attach to labels such as enzymes and nanomaterials that can be used to amplify signals^13^. Sandwich immunosensors based on signals generated from labels have several advantages, such as decreased nonspecific adsorption and increased sensitivity^14^. In general, compared with label-free amperometric immunosensors, sandwich-type amperometric immunosensors result in more sensitive assays with a wide detection range because of signal amplification with detection antibody labels^13,14^. Sandwich-type amperometric immunosensors, which convert immune responses triggered by probe target immunocomplexes into readable current signals, are powerful analytical devices.

Multiwalled carbon nanotubes (MWCNTs) have attracted the attention of researchers in recent decades because of their outstanding electrical conductivity and large specific surface areas; hence, they have been widely applied in catalysis, adsorption and energy storage systems^15,16^. In this work, MWCNTs with sufficient transport channels were employed to enhance the current response of a developed amperometric immunosensor. Metal nanoparticles have attracted interest because of their unique physical, chemical and electronic properties and potential applications in amperometric sensors^17,18^. The excellent conductivity of metal nanoparticles enhances the peak current by transferring electrons from the redox centers in target molecules to the electrode surfaces^19^. Among metal nanoparticles, noble metal nanoparticles (such as gold nanoparticles, platinum nanoparticles and silver nanoparticles), which have good biocompatibility, high catalytic activity and superior electrical conductivity, have been widely used in amperometric immunosensors^13,20^. In addition, bimetallic metal nanoparticles have attracted specific interest because they exhibit distinct unique characteristics, such as enhanced electrical conductivity, high catalytic ability and long-term stability, unlike single metal nanoparticles because of the synergistic effects between bimetallic metal nanoparticles^21,22^. Platinum nanoparticle and silver nanoparticle catalysts have been used in immunosensors in recent years because of their unique catalytic abilities. Platinum silver alloy nanoparticles, which are catalysts with synergistic effects between Ag and Pt nanoparticles, exhibit extraordinary electrocatalytic activity for H_2_O_2_ reduction^23^. In particular, MWCNTs with more active sites and a larger functional surface area were utilized to immobilize Pt/AgNPs to load more active probes bound to biomolecules^24^. Pt/AgNPs can combine with MWCNTs to form composites, which can prevent the agglomeration of Pt/AgNPs and ensure that the nanoparticle size is maintained. Furthermore, MWCNTs have a fast electron transfer rate and a large specific surface area, which can improve immunosensor properties^15^. Inspired by this, bimetallic Pt/AgNPs immobilized on the surface of MWCNTs were developed as a signal amplification label in this work, and the signal was effectively amplified based on the dual catalytic effect of Pt/AgNPs and MWCNTs on H_2_O_2_.

Another crucial factor in obtaining sensitive electrical signals is the immobilization of biomolecules on highly conductive substrates. MWCNTs, which have remarkable electron transfer capability, are promising substrate materials^25^. However, MWCNTs have poor solubility, which is a major barrier to developing immunosensors based on MWCNTs. To optimize the use of MWCNTs for immunosensor applications, it is necessary to functionalize MWCNTs with biomaterials. Among the various biomaterials, chitosan (Chi) is a popular matrix for immobilizing immunosensor elements due to its nontoxicity, excellent film properties, biocompatibility and availability of amine/hydroxyl groups; additionally, Chi can be used to immobilize biomolecules for biosensor applications^26^. However, due to the nonconductive nature of Chi, its use in the preparation of an electrochemical immunosensor has been limited. To increase the conductivity of Chi, a variety of nanomaterials with high conductivity have been incorporated with Chi^27^. Among them, MWCNTs have a low cost, large surface area and conductive properties. More importantly, Chi can covalently attach to MWCNTs via π–π interactions to form MWCNTs-Chi nanobiocomposites. This approach involves the incorporation of MWCNTs with a polymeric backbone, which is helpful for increasing the solubility of MWCNTs without decreasing their conductivity^28^; this approach can not only synergistically promote electron conduction but also effectively immobilize biomolecules such as antibodies and maintain their bioactivity^29^. Based on the above advantages, MWCNTs-Chi nanobiocomposites as substrates provide a suitable microenvironment for immunosensors.

In this study, bimetallic Pt/AgNPs immobilized on the surface of MWCNTs were used as a label to promote dual amplification of the current signal. MWCNTs-Chi were used as a substrate to provide a stable microenvironment for maintaining biomolecular activity and effectively increasing the electron transfer rate on the surface of the electrode. With good coordination of bimetallic Pt/AgNPs immobilized on the surface of MWCNTs-Chi, the established electrochemical immunosensor showed remarkable analytical performance and yielded convincing results in clinical sample assays, demonstrating its good potential for application in disease detection.

## Results and discussion

### Characterization of the nanomaterials

**Figure 1:**
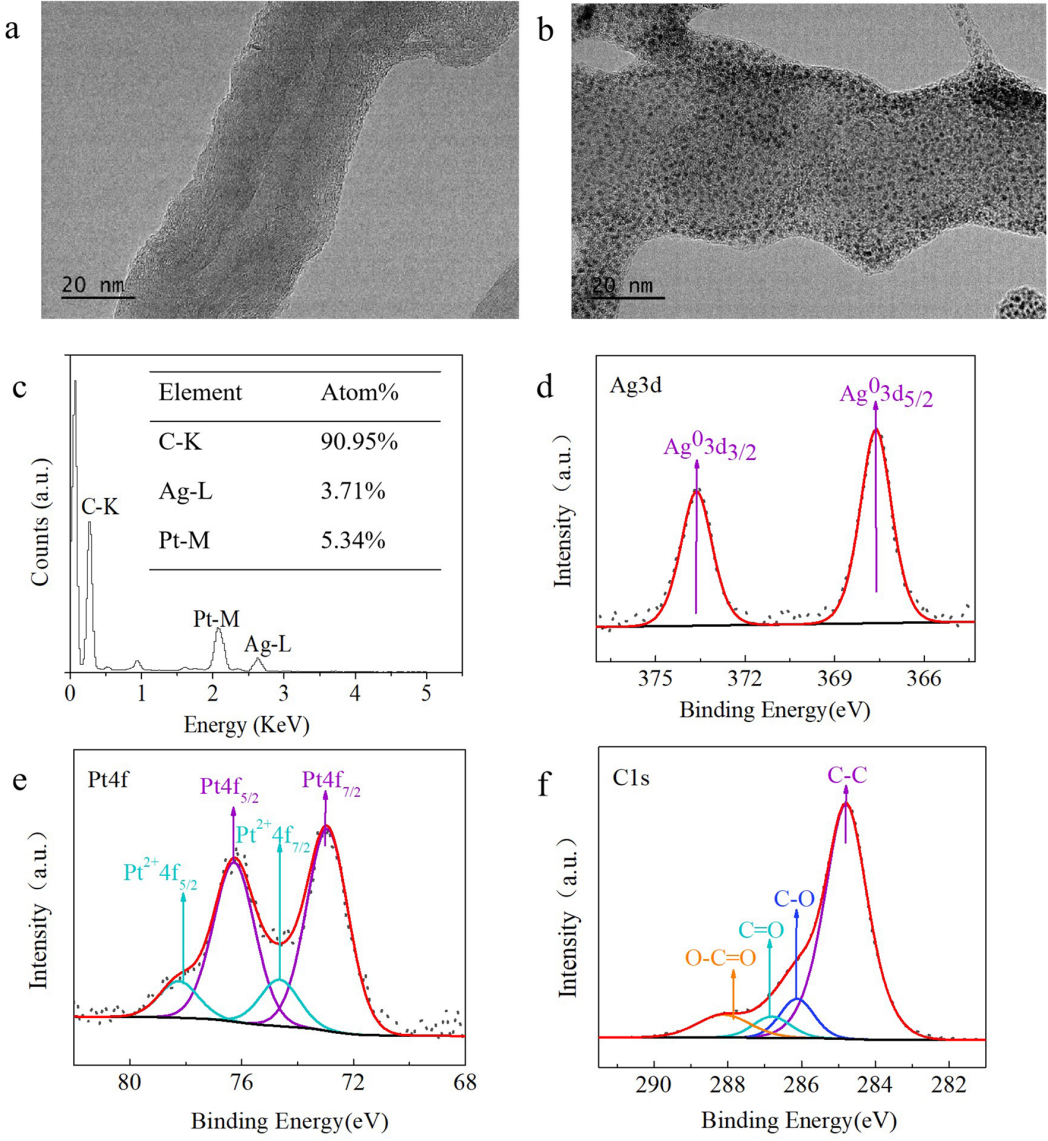
Characterization of the related nanocomposites. TEM images of MWCNTs-Chi (**a**) and MWCNTs-Chi-Pt/AgNPs (**b**); EDS elemental analysis of MWCNTs-Chi-Pt/AgNPs (**c**); High-resolution (**d**) Ag 3d, (**e**) Pt 4f and (**f**) C 1s XPS spectra of MWCNTs-Chi-Pt/AgNPs.

Transmission electron microscopy (TEM) was employed to characterize the features of the MWCNTs-Chi and MWCNTs-Chi-Pt/AgNPs. Figure 1a shows the typical tubular structure of MWCNTs. Figure 1b shows TEM images of the MWCNTs-Chi-Pt/AgNPs. Pt/AgNPs were uniformly distributed on the surface of MWCNTs-Chi. Moreover, energy dispersive X-ray spectroscopy (EDS) was employed to confirm the presence of Pt and Ag in the MWCNTs-Chi-Pt/AgNPs, and the results showed that the Pt/AgNPs were successfully loaded on the surface of the MWCNTs (Fig. 1c). Here, we suspect that Pt^2+^ and Ag^+^ ions in aqueous solution were adsorbed onto the MWCNTs-Chi nanocomposite via chelation and reduced to their corresponding metal nanomaterials by Chi^30,31^. Chi acted as a reducing agent and stabilizing agent. The MWCNTs acted as carriers to load more Pt/AgNPs and prevent the agglomeration of Pt/AgNPs to ensure that their size was consistent with that of the nanoparticles.

X-ray photoelectron spectroscopy (XPS) was employed to survey the surface features of the MWCNTs-Chi-Pt/AgNPs. The XPS spectrum revealed diffraction peaks at 367.62 (3d_5/2_) and 373.63 (3d_3/2_) eV (Fig. 1d), which corresponded well to Ag^0^, and no obvious peak attributed to Ag^+^ was observed, which indicated that metallic Ag^0^ was the predominant species^32^. The Ag 3d peak shifted to a lower value of 367.62 eV relative to that of pure Ag (368.3 eV)^33^ because of the modification of Ag by alloying with Pt. These results confirmed the formation of the Pt/AgNPs alloy during the synthesis of MWCNTs-Chi-Pt/AgNPs^34^.

As shown in Fig. 1e, the binding energies at 72.94 and 76.27 eV corresponded to Pt 4f_7/2_ and Pt 4f_5/2_, respectively, which were attributed to Pt^0^, while the binding energies at 74.64 and 78.25 eV were assigned to Pt^2+^. These results demonstrated that metallic Pt^0^ was formed and that the partial reduction of platinum atoms was incomplete^35^.

The high-resolution C 1 s XPS spectrum of the MWCNTs-Chi-Pt/AgNPs is shown in Fig. 1f. Four diffraction peaks were observed at 284.78, 286.08, 286.7 and 288.13 eV, which were assigned to the covalent C–C, C–O, C=O, and O–C=O bonds, respectively^36^. The diffraction peak intensity ratio of C–C was much higher for MWCNTs-Chi-Pt/AgNPs than for MWCNTs-Chi (Fig. S2), demonstrating the efficient reduction of MWCNTs-Chi^37^.

### Electrochemical characterization of the immunosensor

**Figure 2:**
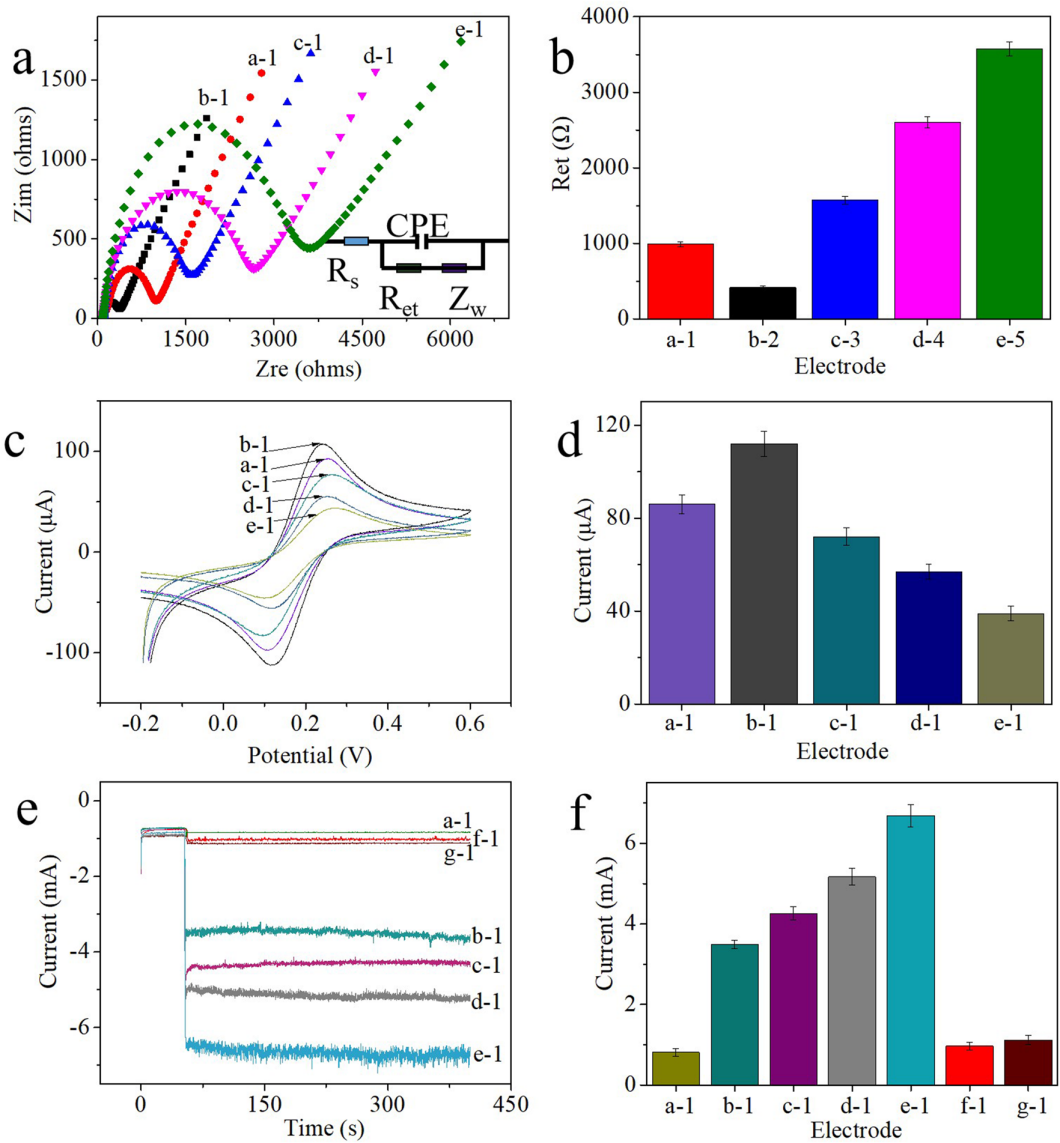
(**a**) EIS Nyquist plots and (**b**) R_et_ values of electrodes at different modification steps in [Fe(CN)_6_]^3−/4−^ (2.5 mM) and 0.1 M KCl mixed solution: (a-1) GCE, (b-1) GCE-MWCNTs-Chi, (c-1) GCE-MWCNTs-Chi-FAdV-I/MAb, (d-1) GCE-MWCNTs-Chi-FAdV-I/MAb-BSA, (e-1) GCE-MWCNTs-Chi-FAdV-I/MAb-BSA-FAdV-I. (**c**) and (**d**) CV curves of the (a-1) GCE, (b-1) GCE-MWCNTs-Chi, (c-1) GCE-MWCNTs-Chi-FAdV-I/MAb, (d-1) GCE-MWCNTs-Chi-FAdV-I/MAb-BSA, and (e-1) GCE-MWCNTs-Chi-FAdV-I/MAb-BSA-FAdV-I. The scan rate was 50 mV s^−1^. (**e** and **f**) Amperometric i-t curves of the immunosensor for the detection of 10^3.43^ EID_50_/mL FAdV-I (a-1) blank; (b-1) MWCNTs-Chi-PtNPs-FAdV-I/PAb; (c-1) MWCNTs-Chi-AgNPs-FAdV-I/PAb; (d-1) Chi-Pt/AgNPs-FAdV-I/PAb; and (d-1) MWCNTs-Chi-Pt/AgNPs-FAdV-I/PAb as labels; (f-1) immunosensor without incubation of FAdV-I/MAb (g-1) without incubation of FAdV-I/MAb but with incubation of FAdV-I and MWCNTs-Chi-Pt/AgNPs-FAdV-I/PAb as labels at -0.1 V toward the reduction of 5 mM H_2_O_2_ in PBS (10 mL, pH 7.4). (Error bar = RSD, n = 5).

In this study, EIS was employed to investigate the step-by-step assembly process of the immunosensor in a mixed solution of [Fe(CN)_6_]^3−/4−^ (2.5 mM) and 0.1 M KCl with an amplitude of 10 mV at 0.2 V and frequencies ranging from 0.01 Hz to 100 kHz. The Nyquist plots obtained by the EIS method were fitted to the effective impedance of the electrode, which is usually expressed as the Nyquist diameter of the semicircle of the graph, which is also known as the electron transfer resistance (R_et_). According to the EIS Nyquist plots, the low-frequency region usually appears as a line that reflects the diffusion process on the electrode surface. The high-frequency region appears as a semicircle that corresponds to R_et_. Figure 2a shows the EIS Nyquist plots obtained during the step-by-step electrode assembly process. In these plots, a semicircle with an R_et_ of 1039 Ω (%RSD) = 3.29 was observed for the bare GCE (Fig. 2a, curve a-1 and b, column a-1), and a smaller semicircle with an R_et_ of 418 Ω (%RSD) = 2.47 was obtained after the GCE was modified with MWCNTs-Chi (Fig. 2a, curve -1 and b, column b-1) because the excellent conductivity of the MWCNTs promoted electron transfer on the surface of the electrode. Then, the electrode was modified by FAdV-I/MAb, BSA and 10^3.43^ EID_50_/mL FAdV-I. The results (Fig. 2b, columns c-1-e-1) showed that the obtained relevant R_et_ values sequentially increased to 1575 Ω (%RSD = 3.14), 2630 Ω (%RSD = 2.91) and 3584 Ω (%RSD = 3.21) because FAdV-I/MAb, BSA and FAdV-I, respectively, have poor conductivity of protein molecules. These results revealed that each step of the electrode modification was successful. The Randles equivalent circuit model is shown in the illustration.

Furthermore, cyclic voltammetry (CV) is another valid technique for verifying the modification of electrodes. The CV data of the GCE electrode demonstrated the effectively reversible redox properties of Fe(CN)_6_^4−/3−^ (Fig. 2c,d, curve and column a-1), and the peak current of the GCE-MWCNTs-Chi increased due to the good conductivity of the MWCNTs (Fig. 2c,d, curve and column b-1). After FAdV-I/MAb, BSA and FAdV-I (10^3.43^ EID_50_/mL) were successively adsorbed on the modified electrode, the corresponding peak currents decreased (Fig. 2c,d, curve and columns c-1–e-1). These results are consistent with the EIS results, indicating that the FAdV-I immunosensor was successfully constructed.

Amperometric i–t curve measurements were used to evaluate the electrocatalytic performance of the developed immunosensor. The results showed that the electrocatalytic activity of MWCNTs-Chi-FAdV-I/MAb-BSA (Fig. 2e,f, curve and column a-1) could be ignored because it was much weaker than that of MWCNTs-Chi-Pt/AgNPs-FAdV-I/PAb (Fig. 2e,f, curve and column e-1). In addition, the electrocatalytic performance of MWCNTs-Chi-PtNPs-FAdV-I/PAb, MWCNTs-Chi-AgNPs-FAdV-I/PAb, Chi-Pt/AgNPs-FAdV-I/PAb, and MWCNTs-Chi-Pt/AgNPs-FAdV-I/PAb as labels during the detection process were compared. The immunosensor using MWCNTs-Chi-Pt/AgNPs-FAdV-I/PAb (Fig. 2e,f, curve and column e-1) as the label had a much higher current change response than did that using MWCNTs-Chi-PtNPs-FAdV-I/PAb (Fig. 2e,f, curve and column b-1), MWCNTs-Chi-AgNPs-FAdV-I/PAb (Fig. 2e,f, curve and column c-1) and Chi-Pt/AgNPs-FAdV-I/PAb (Fig. 2e,f, curve d-1) as the labels. The higher current change response obtained from MWCNTs-Chi-Pt/AgNPs-FAdV-I/PAb was attributed mainly to the excellent catalytic activity of Pt/AgNPs and the excellent electrical conductivity and large specific surface area of the MWCNTs. When the electrode was not immobilized with FAdV-I/MAb and was directly blocked with BSA, the obtained peak current (Fig. 2e,f, curve and column f-1) was similar to the background signal (Fig. 2e,f, curve and column a-1); after it was reacted with 10^3.43^ EID_50_/mL FAdV-I and MWCNTs-Chi-Pt/AgNPs-FAdV-I/PAb, the obtained peak current (Fig. 2e,f, curve and column g-1) was also similar to the background signal. These results indicate that the developed immunosensor has excellent selectivity.

### Optimization of the method

**Figure 3:**
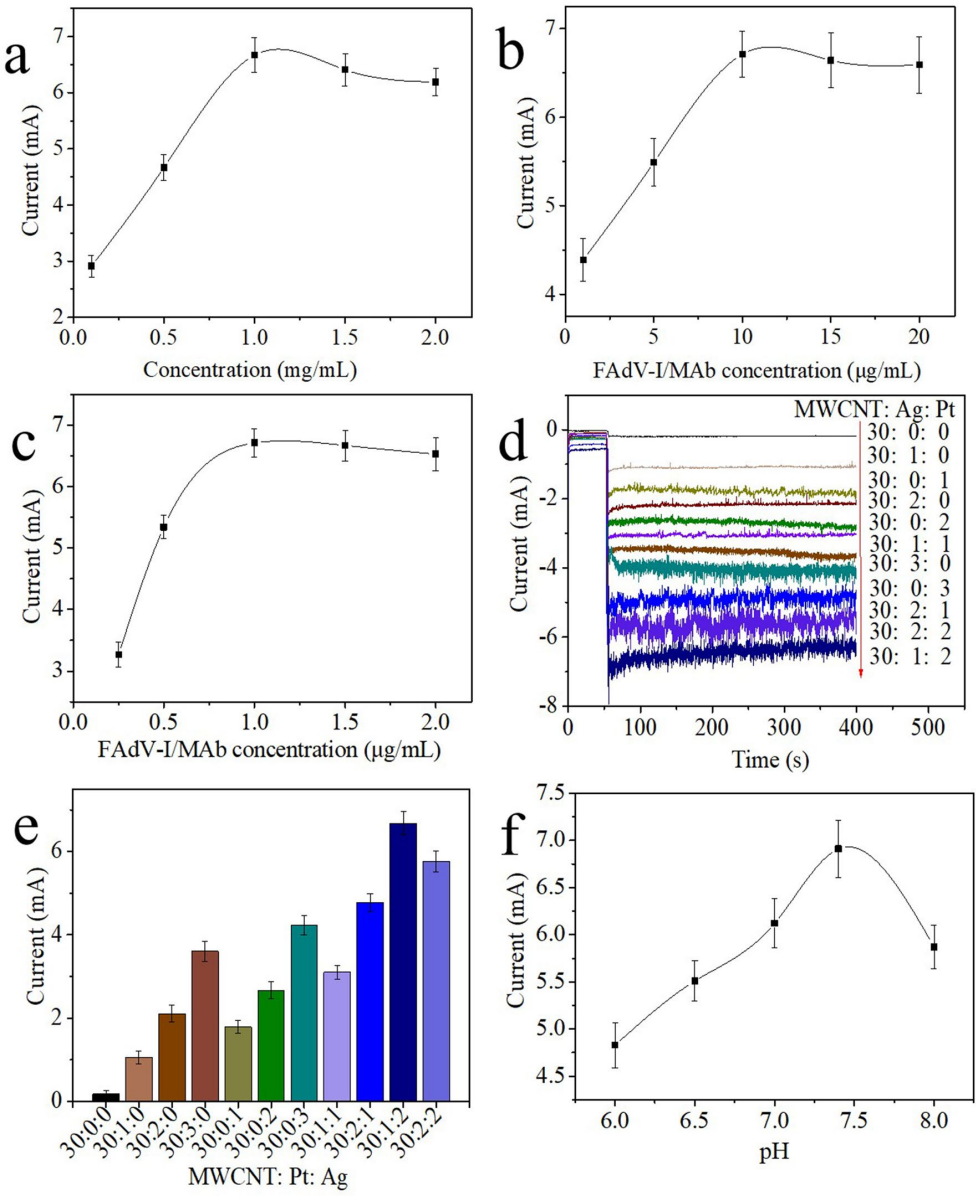
Optimizations of the (**a**) MWCNTs-Chi concentration and (**b**) FAdV-I/MAb concentration. (**c**) FAdV-I/PAb concentration, (**d** and **e**) ratio of MWCNTs to Ag and Pt, and (**f**) pH of the electrolyte. Error bar = RSD (n = 5).

MWCNTs-Chi, which are conductive substrates, are important for enhancing electron transfer between the surface of the electrode and electrolyte and immobilizing FAdV-I/MAb. Hence, the concentration of MWCNTs-Chi was optimized because it affects the amperometric response. GCEs were modified with different concentrations of MWCNTs-Chi to detect 10^3.43^ EID_50_/mL FAdV-I. The currents obtained from the amperometric i–t curve are shown in Fig. 3a. The results showed that the current response increased as the MWCNTs-Chi concentration increased from 0.1 to 1.0 mg/mL. Here, the current response increased due to the increase in the MWCNTs-Chi concentration, directly resulting in an increase in the number of FAdV-I/MAb anchoring sites on the electrode. When the concentration of MWCNTs-Chi increased from 1 to 2 mg/mL, the current response signal reached a plateau. Therefore, 1.0 mg/mL MWCNTs-Chi was the optimal concentration.

The concentrations of FAdV-I/MAb and FAdV-I/PAb were investigated, and the results are shown in Fig. 3b,c. The corresponding response currents gradually increased with increasing FAdV-I/MAb and FAdV-I/PAb concentrations, and the corresponding response currents reached maximum values at 10 µg/mL and 1.0 µg/mL, respectively. As the concentrations of FAdV-I/MAb and FAdV-I/PAb continued to increase, the corresponding response currents reached a plateau, indicating that the concentrations of FAdV-I/MAb and FAdV-I/PAb were oversaturated. Therefore, the optimal concentrations of FAdV-I/MAb and FAdV-I/PAb were 10 µg/mL and 1.0 µg/mL, respectively.

In this work, the MWCNTs-Chi-Pt/AgNPs-FAdV-I/PAb nanocomposite was used as a signal label, and the response current was affected by the ratio of MWCNT, Ag and Pt (MWCNT:Ag:Pt). The results are shown in Fig. 3d,e. When Ag was present, the corresponding signal increased as the ratio of MWCNT to Ag to Pt increased from 30:1:0 to 30:3:0. When Pt was present, the corresponding signal increased as the ratio of MWCNT to Ag and Pt increased from 30:0:1 to 30:0:3. When both Ag and Pt were present, the corresponding signal increased as the ratio of MWCNT to Ag to Pt increased from 30:1:1 to 30:1:2, and the corresponding signal decreased as the ratio of MWCNT to Ag to Pt increased to 30:2:2. Furthermore, the presence of Ag and Pt resulted in a greater catalytic ability for H_2_O_2_ than for single metal nanoparticles because of the synergistic effects between Ag and Pt. Hence, a MWCNT:Ag:Pt ratio of 30:1:2 was the best choice in this study.

The signal of electrochemical immunosensors is usually affected by the pH of the electrolyte. In this work, the pH in the electrolyte was optimized, and the results are shown in Fig. 3f. The corresponding currents increased as the pH increased from 6.0 to 7.4, and the corresponding currents decreased when the pH of the electrolyte was above 7.4. Hence, pH = 7.4 was the optimal electrolyte. The stability of the antigen‒antibody combination may be affected by acidic or alkaline solutions, leading to changes in the signal.

**Figure 4:**
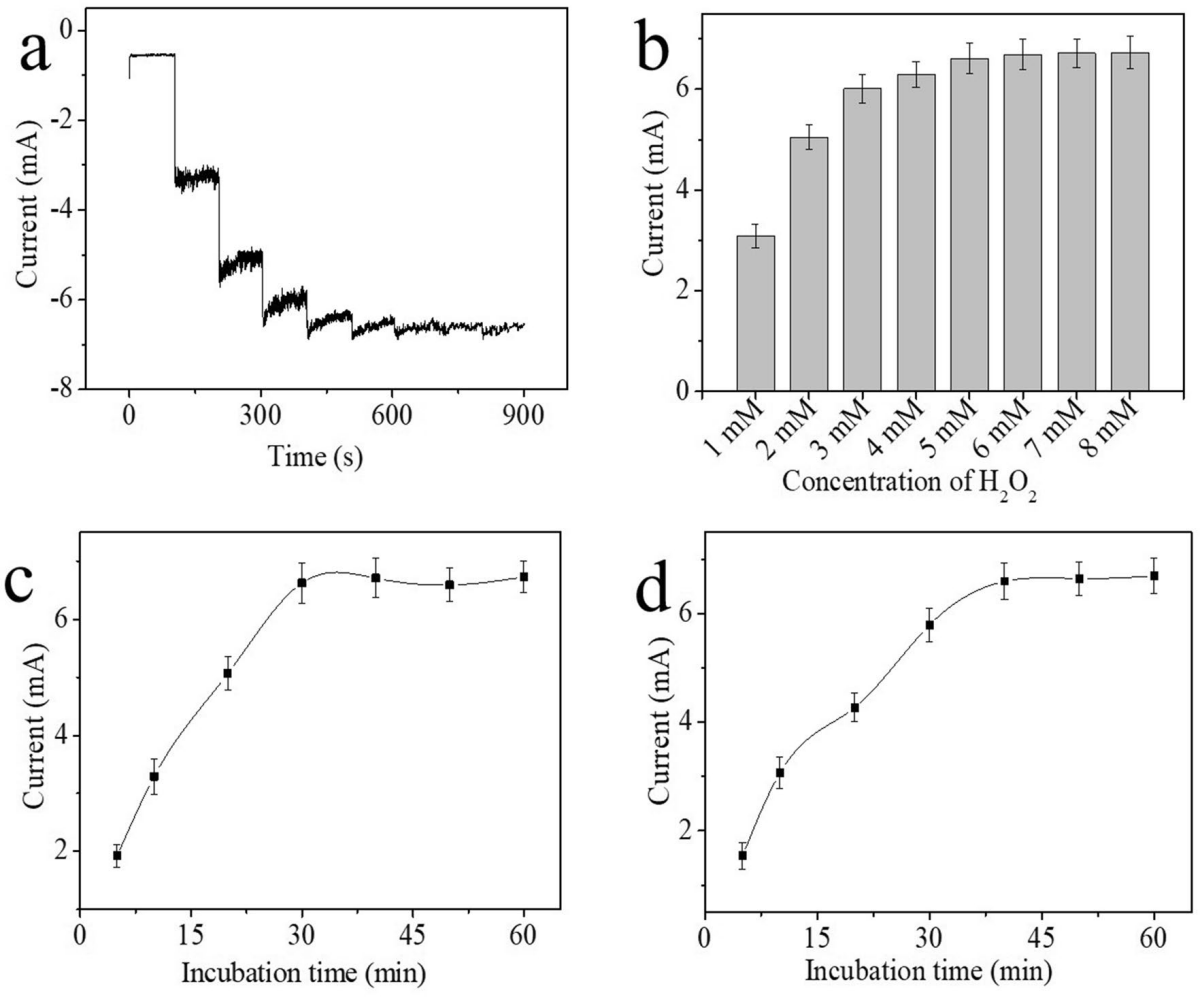
Optimization of (**a, b**) the concentration of H_2_O_2_, (**c**) incubation time of FAdV-I, and (**d**) incubation time of the MWCNTs-Chi-Pt/AgNPs-FAdV-I/PAb nanocomposite. The concentration of the target FAdV-I was 10^3.43^ EID_50_/mL in the optimization experiments. Error bar = RSD (n = 5).

The concentration of H_2_O_2_ was optimized, and the results are shown in Fig. 4a,b. The corresponding currents increased when the concentration of H_2_O_2_ increased from 1 to 5 mM, and the corresponding currents plateaued as the concentration of H_2_O_2_ continued to increase because H_2_O_2_ reached saturation. Hence, 5 mM H_2_O_2_ was chosen as the optimal concentration for addition to the electrolyte.

Furthermore, the incubation time of the sample (FAdV-I) and signal label materials (MWCNTs-Chi-Pt/AgNPs-FAdV-I/PAb nanocomposite) considerably influenced the immunosensor. The corresponding currents increased with increasing incubation time for 30 min and 40 min for FAdV-I and MWCNTs-Chi-Pt/AgNPs-FAdV-I/PAb, respectively, (Fig. 4c,d). The corresponding currents plateaued as the incubation time of FAdV-I and MWCNTs-Chi-Pt/AgNPs-FAdV-I/PAb was extended because the immunoreaction was complete. Hence, the optimal incubation times for FAdV-I and MWCNTs-Chi-Pt/AgNPs-FAdV-I/PAb were 30 min and 40 min, respectively.

### Detection of FAdV-I with the immunosensor

**Figure 5:**
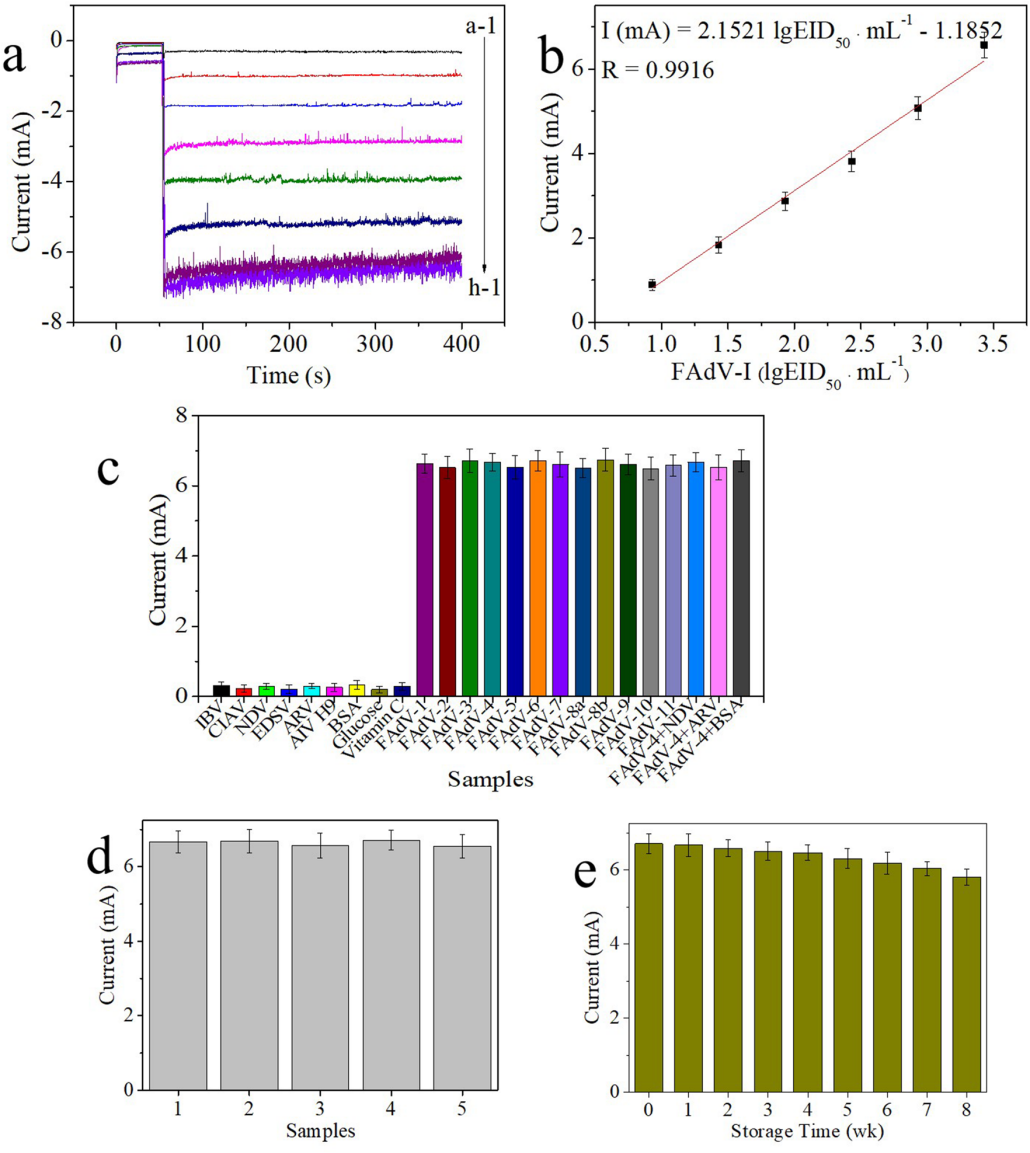
(**a**) Amperometric i-t current response of the developed electrochemical immunosensor for detecting the following concentrations of FAdV-I at − 0.1 V vs. SCE: (a-1) 10^0.43^ EID_50_ mL^−1^, (b-1) 10^0.93^ EID_50_ mL^−1^, (c-1) 10^1.43^ EID_50_ mL^−1^, (d-1) 10^1.93^ EID_50_ mL^−1^, (e-1) 10^2.43^ EID_50_ mL^−1^, (f-1) 10^2.93^ EID_50_ mL^−1^, (g-1) 10^3.43^ EID_50_ mL^−1^, and (h-1) 10^3.93^ EID_50_ mL^−1^. (**b**) Calibration curve of the response of the developed electrochemical immunosensor to different concentrations of FAdV-I. Error bar = RSD (n = 5). (**c**) Specificity of the developed electrochemical immunosensor for the target FAdV-I and other interfering substances. (**d**) Reproducibility of the proposed electrochemical immunosensor in the presence of 10^3.43^ EID_50_ mL^−1^ FAdV-I. (**e**) Long-term stability of the developed electrochemical immunosensor.

The selected immunosensor was used to detect FAdV-I at concentrations ranging from 10^3.93^ to 10^0.93^ EID_50_ mL^−1^ to evaluate its detection capability. Figure 5a shows that the corresponding signal intensity increased with increasing concentrations of FAdV-I. Furthermore, the current signal of the blank (curve a-1) was significantly lower than the current signal of FAdV-I (curves b-1–h-1), indicating that the background current of MWCNTs-Chi-FAdV-I/MAb-BSA and nonspecific adsorption on the surface of the immunosensor were negligible. The calibration plot shown in Fig. 5b shows a good linear relationship between the logarithm values of the FAdV-I concentration and the change in the current signal. The linear regression equation I (mA) = 2.1521 lg EID_50_ mL^−1^—1.1852 (correlation coefficient of R = 0.9916) and a low detection limit (10^0.67^ EID_50_ mL^−1^ (S/N = 3)) were obtained. The excellent analytical performance of the exploited immunosensor was attributed to the following factors: (1) MWCNTs-Chi, which has remarkable electroconductivity and a large specific surface area, were used to modify the electrode and not only promoted electron transfer between the surface of the electrode and electrolyte but also provided enough active sites for FAdV-I/MAb binding; (2) the MWCNTs-Chi-Pt/AgNPs-FAdV-I/PAb nanocomposite had excellent electrocatalytic capability for H_2_O_2_ reduction due to electron–electron correlation assistance; (3) FAdV-I/MAb, which has good specificity, was used as a capture antibody to ensure the specificity of the developed immunosensor; and (4) FAdV-I/PAb, which has more antigen-binding sites as a detection antibody, can enhance the sensitivity of the proposed immunosensor.

### Selectivity, reproducibility and stability of the immunosensor

The specificity of the immunosensor plays an important role in the analysis of clinical samples. Interfering substances that may be present in the clinical sample, including IBV (10^4.79^ EID_50_ mL^−1^), CIAV (10^5.31^ EID_50_ mL^−1^), NDV (10^6.73^ EID_50_ mL^−1^), EDSV (10^5.17^ EID_50_ mL^−1^), ARV (10^7.18^ EID_50_ mL^−1^), AIV H9 (10^6.47^ EID_50_ mL^−1^), BSA (1.0 μg/mL), glucose (1.0 μg/mL) and vitamin C (1.0 μg/mL), were employed to evaluate the specificity of the exploited immunosensor (Fig. 5c). The current responses of the abovementioned interfering substances were similar to those of the blank solution but markedly lower than those of FAdV-I (including 12 serotypes FAdV-1 (10^5.75^ EID_50_ mL^−1^), FAdV-2 (10^4.38^ EID_50_ mL^−1^), FAdV-3 (10^6.21^ EID_50_ mL^−1^), FAdV-4 (10^4.92^ EID_50_ mL^−1^), FAdV-5 (10^5.47^ EID_50_ mL^−1^), FAdV-6 (10^6.35^ EID_50_ mL^−1^), FAdV-7 (10^4.63^ EID_50_ mL^−1^), FAdV-8a (10^6.27^ EID_50_ mL^−1^), FAdV-8b (10^7.35^ EID_50_ mL^−1^), FAdV-9 (10^5.79^ EID_50_ mL^−1^), FAdV-10 (10^6.24^ EID_50_ mL^−1^), and FAdV-11 (10^5.62^ EID_50_ mL^−1^). The current responses of the mixtures of FAdV-4 (10^3.43^ EID_50_ mL^−1^) with possible interfering substances (such as NDV, ARV and BSA) were similar to those of FAdV-I. These results indicated that the applied immunosensor had high specificity for FAdV-I detection.

The reproducibility of the developed immunosensor was evaluated by using different batches of the immunosensor to detect the same concentrations of 10^3.43^ EID_50_ mL^−1^ FAdV-I. The results (Fig. 5d) showed that the intra- and interassay RSD values were all less than 5.0%, as shown by the error bars, and these results indicated that the applied immunosensor had good reproducibility.

The storage life of the developed immunosensor was surveyed by storing GCE-MWCNTs-Chi-FAdV-I/MAb-BSA and MWCNTs-Chi-Pt/AgNPs-FAdV-I/PAb nanocomposites at 4 °C until use. Then, GCE-MWCNTs-Chi-FAdV-I/MAb-BSA were successively incubated with 10^3.43^ EID_50_ mL^−1^ FAdV-I and MWCNTs-Chi-Pt/AgNPs-FAdV-I/PAb nanocomposites. As shown in Fig. 5e, the current response remained at 90.01% of that of the new preparation after storage for 7 weeks. Therefore, the storage life of the developed immunosensor was acceptable.

### Clinical sample analysis

The developed immunosensor was used to analyze 125 clinical samples collected from chickens in small poultry flocks in Guangxi, China, that were suspected to be infected with adenoviruses, and the results were 100% consistent with the results of real-time PCR and 98.3% consistent with the results of loop-mediated isothermal amplification (LAMP), which were both developed by our group^38,39^, to prove that the developed immunosensor can be used for real samples. The results are shown in Table 1. Fifty-nine of the 125 samples (cloacal swab samples) were identified as positive by the proposed immunosensor, which was consistent with the results of real-time PCR, whereas 58 of the 125 samples were identified as positive by LAMP.

**Table 1:** Comparison of real-time PCR, LAMP and immunosensor methods for detecting FAdV-I in chickens in Guangxi, China.

Method	Total number of samples	Number of positive samples	Positivity rate (%)
Proposed immunosensor	125	59	47.2
Real-time PCR	125	59	47.2
LAMP	125	58	46.4

Furthermore, the proposed immunosensor was used to monitor the recovery of cloacal swab samples after treatment with different concentrations of FAdV-I to evaluate its accuracy and practicality. A series of different concentrations of FAdV-I were added by standard addition methods to the six aforementioned clinical samples that were identified as FAdV-I-positive samples; these concentrations were chosen randomly and detected by the fabricated immunosensor. The results are summarized in Table 2. The recoveries, which were calculated as the ratio between the amount of FAdV-I found and the amount added, ranged from 98.37 to 104.41%, with an RSD ranging from 2.63 to 4.71% (n = 5). These results indicated that the fabrication method was appropriate for FAdV-I detection in the samples.

**Table 2:** Results of the recovery of different concentrations of FAdV-I from clinical samples.

No.	Initial FAdV-I concentration in the sample (EID_50_ mL^−1^)	Added amount of FAdV-I (EID_50_ mL^−1^)	Total found		Recovery rate (%) (n = 5)
			Average (EID_50_ mL^−1^)	RSD (%) (n = 5)	
1	107.61	100.00	211.49	3.72	101.86
2	275.42	200.00	487.24	2.85	102.48
3	427.25	400.00	813.75	3.54	98.37
4	531.74	500.00	1059.31	4.71	102.67
5	693.21	600.00	1350.27	2.63	104.41
6	721.35	700.00	1463.79	3.28	102.99

## Conclusions

In this work, an enzyme-free sandwich amperometric immunosensor was developed for the detection of FAdV-I by using MWCNTs-Chi, which has ideal conductivity and efficient antibody immobilization, as the matrix platform and a MWCNTs-Chi-Pt/AgNPs-FAdV-I/PAb nanocomposite, which has excellent electrocatalytic activity toward H_2_O_2_ reduction, as the signal amplification label. The developed amperometric immunosensor showed high specificity, good sensitivity, accuracy, reproducibility and stability and was capable of detecting FAdV-I in clinical samples. More importantly, the developed method not only enables the application of MWCNTs-Chi-Pt/AgNPs nanocomposites in amperometric electrochemical immunosensors but also provides a new enzyme-free method for the determination of other biomolecules in clinical diagnosis.

## Materials and methods

### Reagents and instruments

Chloroplatinic acid hexahydrate (H_2_PtCl_6_·6H_2_O), silver nitrite (AgNO_3_), bovine serum albumin (BSA, 98% purity) and MWCNTs were purchased from Sigma‒Aldrich Chemical Co. (St. Louis, MO, USA). Chi (Mw 1.5 × 10^–5^, deacetylation degree ≥ 90%), NaNO_3_, H_2_SO_4_ and KMnO_4_ were obtained from Guoyao Group Chemical Reagents Co., Ltd. (Shanghai, China). Deionized water (ddH_2_O, ~ 18 MΩ resistance) prepared by a Millipore water purification system was used to prepare the buffers and solutions. All the chemicals were of analytical reagent grade. Phosphate-buffered saline (PBS) was prepared with 10 mmol/L Na_2_HPO_4_, 10 mmol/L NaH_2_PO_4_ and 0.9% NaCl.

### Apparatus

All the electrochemical measurements were conducted with a PARSTAT 4000A instrument (Princeton Applied Research, USA) in this work. A glassy carbon electrode (GCE, Ø = 3 mm), a saturated calomel electrode (SCE) and a platinum wire electrode were used as the working electrode, reference electrode and counter electrode, respectively, composing a standard three-electrode system. Energy-dispersive X-ray spectroscopy (EDS) elemental analysis and transmission electron microscopy (TEM, Tecnai G2 F30 S-TWIN, FIE, USA) were employed to characterize the nanomaterials.

### Viruses and antibodies

Fowl adenovirus group I-monoclonal antibody (FAdV-I/MAb) and fowl adenovirus group I-polyclonal antibody (FAdV-I/PAb) were prepared by our research group^40^. In brief, codon optimization was performed on the Fiber-2 gene sequence, and a sequence fragment encoding a truncated Fiber-2 protein with strong antigenicity was amplified by PCR and cloned into pET-32a(+) to construct the vector for prokaryotic expression. The recombinant protein was purified. Following the immunization of BALB/c mice with the recombinant protein, the serum was collected as a polyclonal antibody. Splenocytes were harvested and fused with SP2/0 cells, and the positive cell lines were screened by indirect enzyme-linked immunosorbent assay (ELISA) and used to prepare monoclonal antibodies. The hybridoma cell line corresponding to FAdV-I/MAb was obtained from the China Center for Type Culture Collection (CCTCC NO: C2022177). FAdV-1, FAdV-2, FAdV-3, FAdV-4, FAdV-5, FAdV-6, FAdV-7, FAdV-8a, FAdV-8b, FAdV-9, FAdV-10 and FAdV-11 were purchased from the China Institute of Veterinary Drugs Control. Chicken infectious anemia virus (CIAV), infectious bronchitis virus (IBV), Newcastle disease virus (NDV), egg drop syndrome virus (EDSV), avian reovirus (ARV) and the avian influenza virus H9 subtype (AIV H9) were collected and stored in our laboratory prior to use.

### Synthesis of MWCNTs-Chi

MWCNTs-Chi was prepared by following the method described above. First, 0.5 g of Chi was added to 100 mL of 1.0% (v/v) acetic acid solution, and the mixture was magnetically stirred at room temperature for 0.5 h. A 0.5 wt% Chi solution was obtained. Second, 100 mg of MWCNTs was added to 100 mL of Chi solution obtained by the above preparation, ultrasonicated for 0.5 h, and continuously magnetically stirred for 48 h at room temperature. A stable MWCNTs-Chi (1 mg/mL) suspension was subsequently obtained.

### Synthesis of MWCNTs-Chi-Pt/AgNPs-FAdV/PAb

MWCNTs-Chi-Pt/AgNPs-FAdV/PAb were prepared by following the method described above. First, 1 mL of AgNO_3_ (10 mmol/L) and 1 mL of K_2_PtCl_4_ (10 mmol/L) were added to the above-prepared MWCNTs-Chi (30 mL) suspension, and the mixture was continuously magnetically stirred for 5 h at room temperature. Second, the mixture was heated in a water bath to 90 °C and kept for 0.5 h for further reaction (Pb^2+^ and Ag^+^ were reduced to Pt/AgNPs by Chi here); then, MWCNTs-Chi-Pt/AgNPs were obtained. Different proportions of MWCNTs-Chi-Pt/AgNPs were prepared by adding different volumes of AgNO_3_ (10 mmol/L) and K_2_PtCl_4_ (10 mmol/L) to 30 mL of the MWCNTs-Chi (1 mg/mL) suspension by the method mentioned above.

Third, the as-prepared MWCNTs-Chi-Pt/AgNPs (10 mL) were mixed with 1 mL of 1 μg/mL FAdV-I/PAb, and the mixture was vibrated in a shaker at 4 °C for 12 h. The product was subsequently washed with PBS (pH 7.4) containing 1 wt% BSA and centrifuged at 10,000 rpm for 5 min, after which the supernatant was discarded to remove the unbound FAdV-I/PAb. Finally, the obtained MWCNTs-Chi-Pt/AgNPs-FAdV/PAb were redispersed into 10 mL of PBS (pH 7.4) containing 1 wt% BSA.

### Fabrication of an electrochemical immunosensor

First, a glass carbon electrode (GCE, Ø = 3 mm) was polished to a mirror surface with 0.05 μm Al_2_O_3_ polishing powder, washed with ddH_2_O and cleaned via ultrasonication in ddH_2_O, CH_3_CH_2_OH and ddH_2_O for 5 min each.

Second, a 0.5 mol L^−1^ H_2_SO_4_ solution was injected with N_2_ for 5 min to remove oxygen, after which the GCE was scanned via cyclic voltammetry (CV) in H_2_SO_4_ solution at a scanning speed of 50 mV/s and a voltage range of − 0.3 ~  + 1.5 V until the CV current was stable. Afterward, the GCE was washed with ddH_2_O three times and dried with N_2_ for further modification.

Third, 10 μL of 1 mg/mL MWCNTs-Chi was dropped on the surface of the GCE and dried at 37 °C. Next, 10 μL of 10 μg/mL FAdV-I/MAb was deposited onto the electrode, incubated at 4 °C for 8 h, and then washed with PBS (pH 7.4) three times to remove the unbound FAdV-I/MAb.

Fourth, 10 μL of 1 wt% BSA, which was used as a nonspecific binding blocker, was deposited onto the electrode and incubated for 1 h at 37 °C to eliminate nonspecific binding, after which the modified electrode was washed with PBS (pH 7.4) three times to remove the excess BSA. The obtained immunosensor (GCE-MWCNTs-Chi-FAdV-I/MAb-BSA) was stored at 4 °C until use.

### Detection of the electrochemical immunosensor

**Figure 6:**
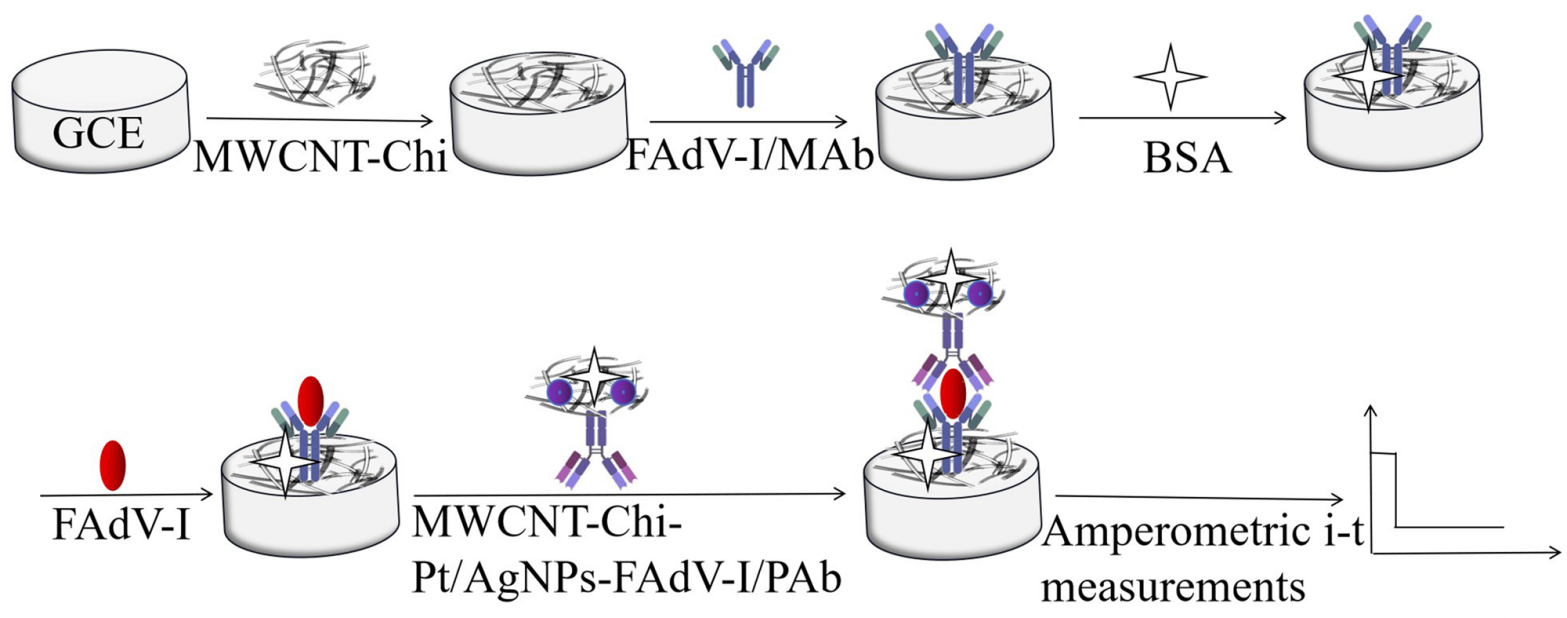
Schematic illustrating the exploited electrochemical immunosensor.

The procedure for the electrochemical immunosensor preparation is shown in Fig. 6. First, 10 μL of solution containing different concentrations of FAdV-I was incubated with the developed immunosensor for 30 min to ensure specific binding of FAdV-I to FAdV-I/MAb, after which the mixture was washed with PBS (pH 7.4) three times to remove unbound FAdV-I and other interfering substances. Second, 10 μL of the developed immunosensor was incubated with MWCNTs-Chi-Pt/AgNPs-FAdV/PAb at 37 °C for 40 min and then washed with PBS (pH 7.4) three additional times to remove the excess MWCNTs-Chi-Pt/AgNPs-FAdV/PAb. Finally, amperometric i–t measurements were performed, and the voltage was set to − 0.1 V based on minimized interfering components and background current (supplementary material Fig. S1). After the signal stabilized (approximately 50 s later), H_2_O_2_ (5 mmol/L) was added to PBS (10 mL, pH 7.4) under continuous stirring, and the current change was recorded. The mechanism of signal production by catalytic H_2_O_2_ was as follows^41^: H_2_O_2_ + e^−^ → OH_ad_ + OH^−^; OH_ad_ + e^−^ → OH^−^; 2H^+^ + 2OH^−^ → 2H_2_O. In the H_2_O_2_ reduction process, the key step is OH_ad_ formation, which helps to amplify the signal for sensitive detection of FAdV-I, and MWCNTs-Chi-Pt/AgNPs can provide abundant catalytic active centers for OH_ad_ adsorption^42^. Electrochemical impedance spectroscopy (EIS) was performed in a [Fe(CN)_6_]^3−/4−^ (2.5 mM) and 0.1 M KCl mixed solution with an amplitude of 10 mV at 0.2 V (open circuit potential) and frequencies ranging from 0.01 Hz to 100 kHz.

### Ethics statement

All the authors confirm that the use of animals in this work was strictly in accordance with the ARRIVE guidelines. This work was approved, conducted and supervised by the Animal Ethics Committee of the Guangxi Veterinary Research Institute. Oral and cloacal swab samples, which were gently collected from chickens in small poultry flocks in Guangxi, China and were suspected to be infected with adenoviruses, were used as clinical samples. The chickens were not anesthetized before sampling, and they were returned to the cages and observed for 0.5 h after sampling. All the authors confirm that all the methods were performed in accordance with the relevant guidelines and regulations.

### Supplementary Information

Supplementary Information.

## Data Availability

All the data generated or analyzed during this study are included in this article and the supplementary material.
