# Supplementary material for: A sandwich amperometric immunosensor for the detection of fowl adenovirus group I based on bimetallic Pt/Ag nanoparticle-functionalized multiwalled carbon nanotubes
Source: Sci Rep. 2024 Jan 2;14:261. doi: 10.1038/s41598-023-50821-x (PMC10762159; doi:10.1038/s41598-023-50821-x)
Supplement: Supplementary file 1 — Supplementary Information. [file 41598_2023_50821_MOESM1_ESM.docx]

Supplementary material

A Sandwich Amperometric Immunosensor for the Detection of Fowl Adenovirus Group I Based on Bimetallic Pt/Ag Nanoparticle-Functionalized Multiwalled Carbon Nanotubes

Jiaoling Huang, Zhixun Xie^*^, Sisi Luo, Meng Li, Liji Xie, Qing Fan, Tingting Zeng, Yanfang Zhang, Minxiu Zhang, Zhiqin Xie, Sheng Wang, Dan Li, You Wei, Xiaofeng Li, Lijun Wan, Hongyu Ren

Guangxi Key Laboratory of Veterinary Biotechnology, Key Laboratory of China (Guangxi)-ASEAN Cross-border Animal Disease Prevention and Control, Ministry of Agriculture and Rural Affairs of China, Guangxi Veterinary Research Institute, Nanning, Guangxi, China

***Corresponding author**

1. mail: xiezhixun@126.com

**Cyclic voltammetry of MWCNTs-Chi-Pt/AgNPs-FAdV/PAb**

Cyclic voltammetry was used to survey the electrocatalytic mechanism of the developed sandwich amperometric immunosensors. Fig. S1 shows the cyclic voltammograms of the developed sandwich immunosensor for the detection of 10^3.43^ EID_50_/mL FAdV-I using MWCNTs-Chi-Pt/AgNPs-FAdV/PAb as a reference in an electrolyte (PBS) at pH=7.4 before (Fig. S1 a) and after (Fig. S1 b) the addition of 5 mM H_2_O_2_. The cyclic voltammetry data of the developed sandwich amperometric immunosensor did not show any obvious reduction peak (Fig. S1 a) before the addition of H_2_O_2._ After the addition of H_2_O_2,_ an obvious increase in the reduction current was obtained at -0.1 V (Fig. S1 b). The results revealed the good electrocatalytic performance of the developed sandwich amperometric immunosensors for the reduction of H_2_O_2_ and revealed that amperometric i-t measurements carried out in an electrolyte (PBS) at pH=7.4 and a potential of -0.1 V were good for decreasing the background current.


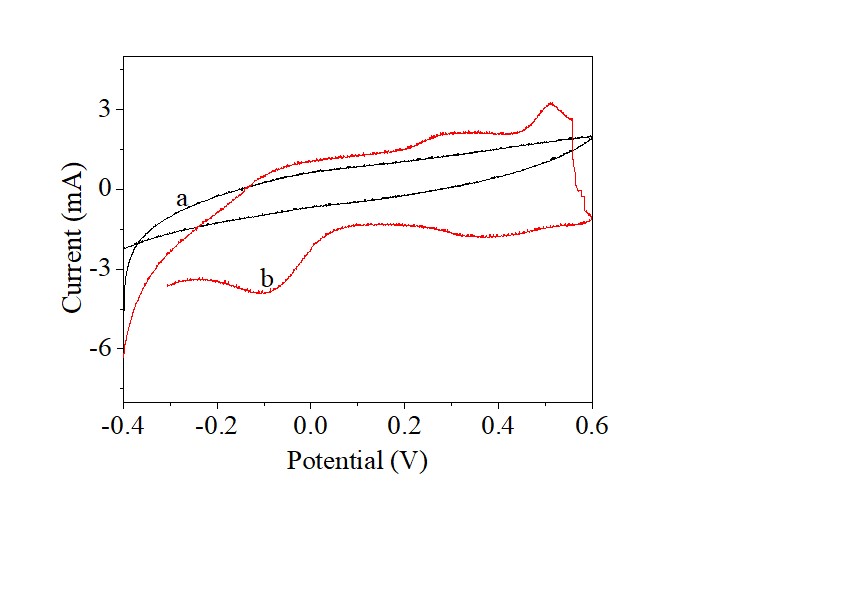


Fig. S1. Cyclic voltammetry (CV) curves of the developed immunosensor in electrolyte (PBS) at pH=7.4 before (a) and after (b) the addition of 5 mM H_2_O_2_.

Fig. S2. High-resolution C 1s XPS spectrum of MWCNTs-Chi.
